# A *cis*-Regulatory Signature for Chordate Anterior Neuroectodermal Genes

**DOI:** 10.1371/journal.pgen.1000912

**Published:** 2010-04-15

**Authors:** Maximilian Haeussler, Yan Jaszczyszyn, Lionel Christiaen, Jean-Stéphane Joly

**Affiliations:** INRA group, UPR3294, Institute of Neurosciences Alfred Fessard, CNRS, Gif-sur-Yvette, France; New York University, United States of America

## Abstract

One of the striking findings of comparative developmental genetics was that expression patterns of core transcription factors are extraordinarily conserved in bilaterians. However, it remains unclear whether *cis*-regulatory elements of their target genes also exhibit common signatures associated with conserved embryonic fields. To address this question, we focused on genes that are active in the anterior neuroectoderm and non-neural ectoderm of the ascidian *Ciona intestinalis*. Following the dissection of a prototypic anterior placodal enhancer, we searched all genomic conserved non-coding elements for duplicated motifs around genes showing anterior neuroectodermal expression. Strikingly, we identified an over-represented pentamer motif corresponding to the binding site of the homeodomain protein OTX, which plays a pivotal role in the anterior development of all bilaterian species. Using an *in vivo* reporter gene assay, we observed that 10 of 23 candidate *cis*-regulatory elements containing duplicated OTX motifs are active in the anterior neuroectoderm, thus showing that this *cis*-regulatory signature is predictive of neuroectodermal enhancers. These results show that a common *cis*-regulatory signature corresponding to K50-*Paired* homeodomain transcription factors is found in non-coding sequences flanking anterior neuroectodermal genes in chordate embryos. Thus, field-specific selector genes impose architectural constraints in the form of combinations of short tags on their target enhancers. This could account for the strong evolutionary conservation of the regulatory elements controlling field-specific selector genes responsible for body plan formation.

## Author Summary

Regional identity in embryos is defined by a few specific transcription factors that activate a large number of target genes through binding to common tags in regulatory sequences. In chordates it is unclear if such tags can be identified in the *cis*-regulatory regions of regionally expressed genes. To address this question we focused on the anterior nervous system where *Otx* codes for a transcription factor that triggers expression of many other head-specific genes. We analyzed an element that is active in the region bordering the anterior nervous system in the marine invertebrate *Ciona intestinalis*. We found that the crucial binding sites have to be duplicated and close enough. One of the pairs is bound by OTX. We showed that anterior nervous system genes are often flanked by duplicated OTX binding sites. We confirmed by transgenic assays that about half of these genomic sequences are active and drive expression anteriorly. This study unravels a simple regulatory logic in the anterior enhancers. It indicates that although there are major changes in the organization of the binding sites at short evolutionary range, conserved expression patterns are partly generated by a duplicated organization of conserved binding sites for region-specific transcription factors.

## Introduction

The concept of “selector genes” was introduced 30 years ago by Garcia-Bellido to define genes that interpret a transient regulatory state and specify the identity of a given developmental field [1]. The question of how embryos execute distinct and unique differentiation programs using these selector genes can be tackled by focusing on how gene expression is encoded in *cis*-regulatory elements and their field-specific *trans*-acting factors (TF).

This concept was more recently extended to terminal selector genes that coordinate the expression of differentiation genes to determine a given cell type [2]. In vertebrates, examples include the *Crx* TF that interacts with another TF to control the expression of target genes in rod photoreceptors [3]–[5]. In vertebrates as well as in flies, *Crx* and its *Drosophila* homolog *Otd* act through a small *cis*-regulatory motif overrepresented in the elements flanking the target genes [6]–[10]. In addition to this evolutionary conserved network, many others in *Caenorhabditis elegans* and *Drosophila melanogaster* have shown that cell specific enhancers contain a common “tag” corresponding to a specific *cis*-regulatory motif, and that this motif is linked to one or a few terminal selector genes [11], [12]. In contrast, during early development, very few studies have reported how a set of region-specific *cis*-regulatory elements responds to field-specific selector genes. In insects, one of the best characterized sets of functionally related *cis*-regulatory elements responds to the gradient of nuclearized *dorsal* TF in the early *Drosophila* embryo [13], [14]. However, the regulatory mechanism of dorsal-ventral patterning is not enough conserved in chordates to allow comparative studies of the regulatory network.

A more general character of bilaterians is the tripartite organization of the nervous system along the antero-posterior axis [15]. In the posterior part (hindbrain and nerve cord), *Hox* genes are expressed in a colinear order. In the domain anterior to the *Hox* genes, several striking similarities in the relative expression patterns of other transcription factors have been noted in bilaterians [16]–[18]. The *OTX*-like homeobox transcription factors (*otd* in insects) are expressed in the anteriormost part of animals as diverse as cnidarians, insects, annelids, urochordates and vertebrates [19]–[21]. In chordates, OTX has a sustained expression in the anterior neuroectoderm and in derivatives of anterior ectoderm such as placodes, stomodeum [20], [22]. In mice, null-mutants of this gene lack various head structures [23]. These results suggest that OTX-like proteins belong to a conserved developmental control system operating in the anterior parts of the brain, different from the one encoded by the *Hox* complexes [24].

Many homeodomain proteins bind to the core DNA sequence ATTA, but several subfamilies have longer binding specificities around this core [25], [26]. OTX homeodomain proteins contain a lysine at position 50 which confers them additional specificity to guanines 5′ of the ATTA motif, resulting in a core recognition sequence of GATTA/TAATC [27]. The DNA binding domains of homeobox gene families are highly similar over large evolutionary distances and cross-species experiments have demonstrated that the OTX proteins can be exchanged between flies, mice and human without major developmental defects [28], [29], and more recently between ascidians and mice [24], [30].

For studies of anterior nervous system development, the ascidian *Ciona intestinalis* offers the advantage of a simple chordate body plan with the canonical tripartite brain along the antero-posterior axis [31]. In addition, the genome is small, with short intergenic regions which can be aligned with another ascidian species, thus simplifying the identification of *cis*-regulatory elements [32]. Moreover, complete expression patterns have been determined for thousands of genes and are readily available in public databases [33]–[35]. Therefore, *Ciona intestinalis* constitutes an ideal model system for combining whole genome bioinformatics and experimental *cis*-regulatory analyses.

Here, we first focus on one single anterior ectodermal enhancer in *Ciona intestinalis*. Its detailed analysis points to an internal tandem-like structure and underscores the key role of the selector gene *Otx*. We then examine if other duplicated putative binding sites for OTX preferentially flank anteriorly expressed genes in the genome.

## Results/Discussion

### D1 mediates the initiation of *Ci-pitx* expression in the anterior neural boundary (ANB)

**Figure 1:**
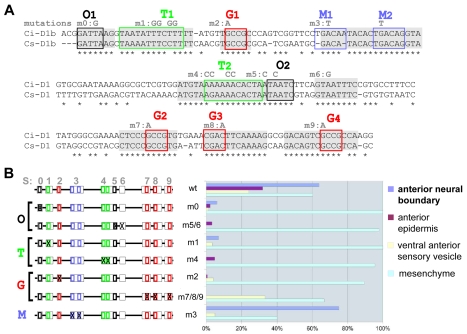
Mutational analysis of D1(bcde) reveals at least three binding sites necessary for its activity. (A) Alignment of D1(bcde) (195pb) between Ci/Cs. Conserved blocks in D1(bcde) with putative transcription factor binding sites (TFBS). Four classes of putative TFBS within ten blocks of conserved sequence: O1 and O2 display the characteristic GATTA/TAATC core sequence that is known to bind K50 *Paired*-related homeodomain proteins, including otx/otd, pitx and goosecoid. Putative Fox protein binding sites were identified in the blocks called T1 and T2 (T/A-rich). Blocks M1 and M2 show similarity to known binding sites of the TALE-class Meis/TGIF homeodomain proteins. Finally, putative Smad1/5/8 binding sites were observed in blocks labelled G1, G2, G3 (G/C-rich). Conserved blocks of nucleotides are highlighted in grey. Putative binding sites are represented by colored boxes. Mutations designed to disrupt the binding specificity of each sites are indicated in grey above the sequence (m0 to m9). (B) Schematic representation of D1(bcde) and series of mutations introduced into the pD1bcde:CES2 construct. On the right is shown for each construct the rate of mid-tailbud embryos expressing the lacZ reporter in the anterior neural boundary (anb), the anterior epidermis (ae), the ventro-anterior sensory vesicle (vasv) and the mesenchyme (mes) (wt n = 444; m0 n = 198; m1 n = 56; m2 n = 348; m3 n = 40; m4 n = 58; m5/6 n = 182; m7/8/9 n = 29). Most mutations led to a general reduction of the expression level in all of aforementioned domains, while mesenchyme expression remained high. Only construct m3 (block M1 and M2) retained the activity in the anterior neural boundary.

**Figure 2:**
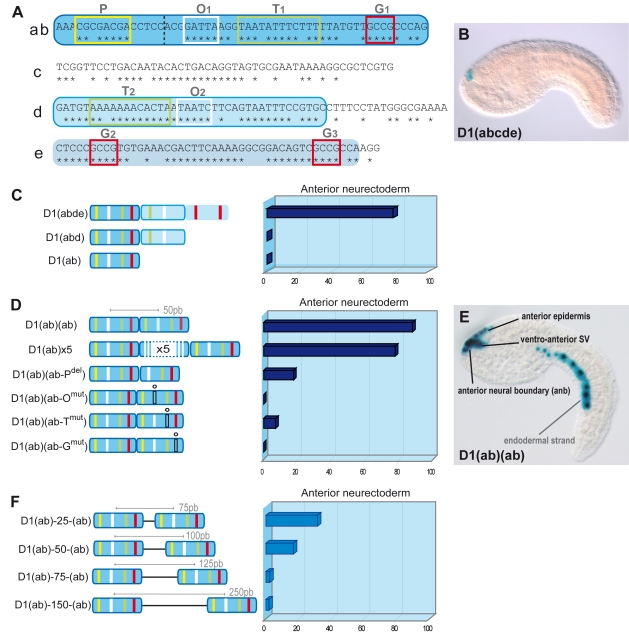
Artificial enhancer constructs reveal a tandem-*like* structure. (A) D1(abcde) *Ci-Pitx* enhancer. Stars show conserved positions with *Ciona savignyi*. The element has been divided into five parts (a, b, c, d, e). (ab) fragment is in dark blue, (d) in light blue with blue outline, (e) in light blue. Conserved nucleotides stretches contain putative transcription factor binding sites (TF-BS). O1 and O2 sites (white box) correspond to the BS for K-50 *Paired* homeodomain proteins and P (yellow), T1, T2 (green) and G1, G2, G3 (red) resemble Pax, Forkhead and Smad protein consensus BS respectively. (B) Side view of an early-tailbud embryo electroporated with pD1abcde:CES2:lacZ: expression in the anterior neural boundary (ANB). In some cases, ectopic expression occurs in the mesenchyme and the tail muscles (not shown). (C) Expression of artificial enhancers in the anterior neuroectoderm of mid-tailbud embryos. LacZ expression was observed after two hours of staining. The D1(abde) construct drives lacZ expression in the anterior neuroectoderm (ANB), ventro-anterior sensory vesicle (vasv) and epineural epidermis (ene) in 77.9% of developed embryos (n = 57). Deletions of (e) or (de), but not (c), abolish LacZ expression (D1(abd), D1(ab)). (D) Two (D1(ab)(ab)) or five (D1(ab)×5) copies of the 54bp D1(ab) drive expression in most of the embryos (88% (n = 167) and 77% (n = 72), respectively). Only 17% of the embryos express lacZ following the deletion of the second P site (D1(ab)(ab-P_del_), n = 90). Mutations of O, T, and G sites in the second copy of (ab) strongly decrease lacZ expression. (D1(ab)(ab-O^mut^): 0% (n = 137), D1(ab)(ab-T^mut^): 7% (n = 84), D1(ab)(ab-G^mut^): 0% (n = 118)). (E) Mid-tailbud embryo electroporated with pD1(ab)(ab):CES2:lacZ. Expression is visible in the ANB, the vasv, the ene and less frequently in the endodermal strand. (F) Introduction of spacer regions of 25/50/75/150 bp between the two D1(ab) fragments strongly decreased the activity of the tandem constructs. From 88% (D1(ab)(ab)) to 31.6% (n = 76), 16.9% (n = 154), 2.5% (n = 79) and 1.9% (n = 106), respectively. Scores were obtained after one week of LacZ revelation.

We have previously described an enhancer sequence (called “D1”, 323bp) that controls expression of the *Ciona intestinalis Pitx* gene in a sub-region overlapping the neural and the non neural ectoderm called the anterior neural boundary (ANB) [36]. For the sake of simplicity, and although ANB has a dual origin, we label it as a derivative of the neuroectoderm and call the region composed of anterior epidermis, ventro-anterior sensory vesicle and ANB, the “anterior neuroectoderm”. For this study, we used a minimal 206 bp fragment of D1 that is sufficient to drive reporter gene expression in the ANB and divided it into five parts (D1a-e, Figure 1A and Figure 2A) for further analysis. Deletion of the first 16pb (D1a, Figure 2A) resulted in the D1bcde fragment (Figure 1A) and led to ectopic reporter gene expression in the anterior epidermis (ae) and ventro-anterior sensory vesicle (vasv) in addition to the expected expression in the ANB. All these elements indicate that D1 responds to neuroectodermal *trans*-activating factors that are not restricted to the ANB and that D1a contains motifs bound by a repressor factor that restricts D1 expression to the sole ANB.

We tested whether D1bcde controls the onset of *Ci-pitx* expression in the ANB. Endogenous *Ci-Pitx*-gene expression was not detected in ANB cells before the initial tailbud stage [37], [38], suggesting that it starts at this stage. To test whether D1bcde recapitulates the temporal pattern of *Ci-Pitx* expression, we assayed reporter gene expression by either X-gal staining or lacZ *in situ* hybridization on the same batch of electroporated embryos fixed at successive stages. The rationale is to take advantage of the delay in β-galactosidase protein synthesis (e.g. [39]), which should produce a marked difference between X-gal and *in situ* staining shortly after the onset of reporter gene expression. We could detect neither lacZ RNAs nor β-galactosidase activity *before* the initial tailbud stage. At this stage, however, *lacZ* transcripts could be detected in 55.4% (n = 46 of N = 71) of the embryos while only 7% (n = 5 of N = 83) showed positive ANB cells after X-gal staining (Table S1). Hence, D1bcde-driven transcription starts at the same time as the endogenous *pitx* gene, which indicates that the D1bcde enhancer element triggers the initiation of *Ci-pitx* expression in ANB cells.

### Short blocks of conserved nucleotides are required for D1 enhancer activity

Conservation between *Ciona intestinalis* and *savignyi* genomic sequences is not uniformly distributed throughout conserved non coding elements (CNEs) but rather concentrated in short blocks of identical nucleotides, which point to candidate transcription factor binding sites (TF-BS; Figure 1A, Figure 2A). We identified four classes of putative TF-BS based on nucleotide composition and by querying binding site databases [40], [41]. One of them matches the OTX/K-50 *paired* homeodomain consensus sequence (sites O1 and O2, Figure 1A and Figure 2A). Other sites, called T (T/A-rich), G (G/C-rich) and M, bear resemblance to Forkhead, Smad and Meis family factors, respectively (Figure 1A). Some of them (P, T1, T2 were not completely conserved in the genome alignment. But each class of these candidate binding sites was represented at least twice in the minimal D1bcde element. The function of these candidate TF-BS was tested by introducing point mutations in the corresponding blocks of conserved sequences, followed by reporter gene expression assays. With the exception of mutations disrupting the “M” sites, each one of the individual modifications of O, T and G sequences reduced reporter gene expression in the anterior neuroectoderm derivatives (Figure 1B). Taken together, these observations indicate that D1 enhancer activity requires at least two copies of each one of three distinct classes of conserved putative TF-BS (Figure 1).

### A tandem organization of binding sites is required for D1 activity

The aforementioned observation that the essential putative binding sites occur several times in the enhancer led us to investigate whether the structure of D1 bears functional significance to its enhancer activity. Notably, the 54-bp D1(ab) element (Figure 2A) contains the three previously mentioned conserved motifs O, T and G in addition to a putative Pax binding site (P), but D1(ab) is not sufficient to enhance reporter gene transcription (Figure 2C). Since each of the critical sites is represented at least twice in the full length enhancer, we asked whether D1 enhancer activity relies on this tandem-like repetition of essential binding sites. We created artificial enhancers containing multiple copies of D1(ab) and found that as little as two copies of D1(ab) were sufficient to drive strong lacZ expression in the anterior neuroectoderm (88% of 167 tailbud embryos (Figure 2D and 2E)).

To test whether enhancer activity of the D1(ab) dimer relies specifically on the duplication of O, T and G sites, we introduced point mutations in the second D1(ab) copy. Each of these mutations strongly reduced enhancer activity (Figure 2D). These observations are reminiscent of the requirement for multiple copies of *bicoid* binding sites for target gene activation during *Drosophila* head development [42] and the general tendency of binding sites to occur in clusters [43]. Our results demonstrate that duplications of critical binding sites are essential for D1 enhancer activity and do not constitute mere redundancy.

We next asked whether the distance between the duplicated 54bp elements influenced the activity of the artificial D1(ab) dimer. To this aim, we designed sequences that are not predicted to bind any characterized transcription factors from the Uniprobe database (see Materials and Methods) and inserted 25, 50, 75 and 150bp spacers between the D1(ab) duplicates. Overall, enhancer activity of these constructs is reduced compared to the original D1(ab) dimer and almost completely abolished with the 75bp and 150bp spacers (Figure 2F). Similar structural constraints were reported in the *Drosophila knirps* enhancer, which was shown to require a specific arrangement of duplicated *bicoid* binding sites for activation [44], [45]. Similarly, *even-skipped* enhancers contain a conserved structure of paired binding sites [46] and duplicated and relatively distant (30–200bp) TFBS are necessary for a correct activity of the SV40 enhancer [47] and the *lac* operon [48]. Taken together, our observations demonstrate that D1 enhancer activity relies on the clustering of duplicate short conserved sequences.

### *Ci-Otx* affects D1 enhancer activity

Among D1(ab) essential putative binding sites, the GATTA/TAATC “O” sequences correspond to the consensus for K50-*Paired* homeodomain proteins. In ascidians, this family includes Goosecoid, Pitx and Otx. Only Otx, is expressed in the right time and place to account for D1 enhancer activation in the anterior neuroectoderm in *Ciona* [20] and there is only one *Otx* gene in the *Ciona intestinalis* genome.

**Figure 3:**
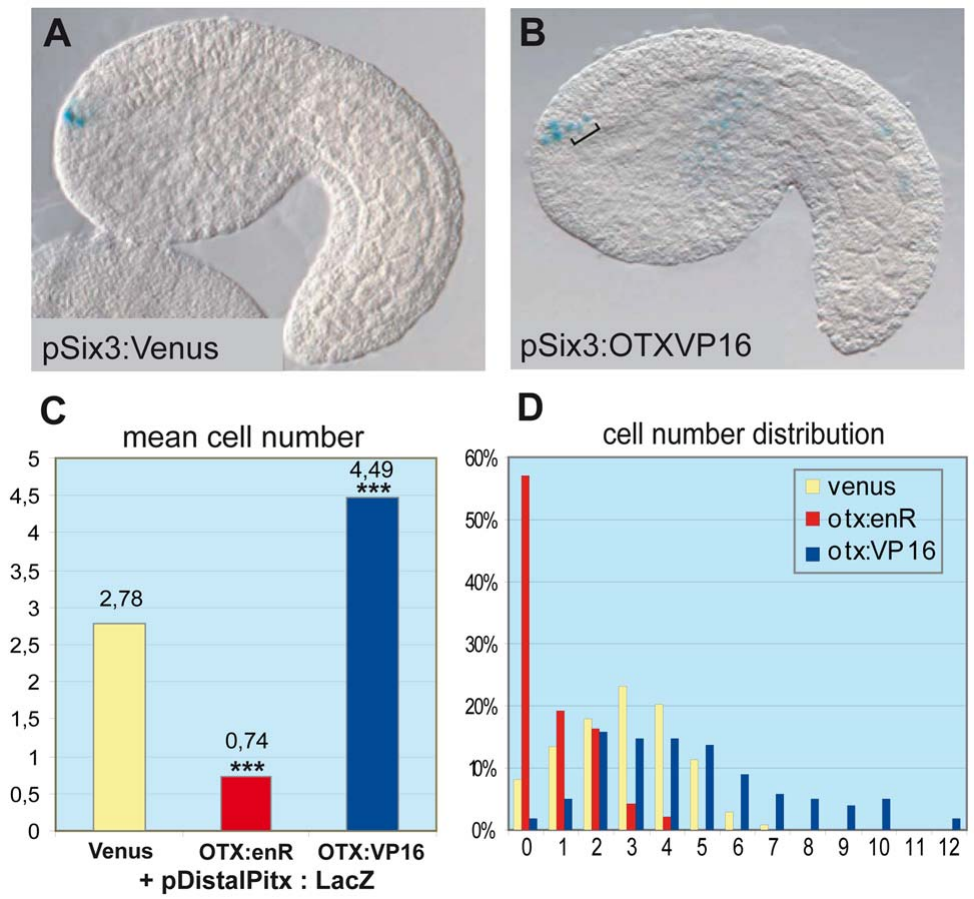
OTX fusions influence the activity of the *Ci-pitx cis*-regulatory element. Co-electroporation of *Pitx* full length distal region (pDistal*Pitx*:lacZ, 5.3kb, containing D1), respectively with pSix3:Venus (control), with pSix3>OTX_HD_::enR (dominant negative OTX) and pSix3>OTX_HD_::VP16 (hyper-active OTX). (A) Side view of an embryo co-electroporated with pDistal*Pitx*:lacZ and pSix3:Venus. Three positive cells can be detected in the ANB. (B) Co-electroporation of pDistal*Pitx*:lacZ and pSix3>OTX_HD_::VP16. In addition to the expression in the ANB, ectopic expression is detected in the ASV cells (bracket) where OTX:VP16 is produced under the control of p*Six3*. (C) Numbers of lacZ expressing cells decrease with the OTX_HD_::enR protein (2.78 to 0.74 cells) and increase with the OTX_HD_::VP16 protein (2.78 to 4.49 cells) The distributions differ significantly from the control in both groups according to two *Wilcoxon–Mann–Whitney* two-sample rank-sum tests: Control/OTXenR (U_OTXenR_ = 3891, n(emb)_OTXenR_ = 139, n(emb)_ctrl_ = 131, P = 2.536e-07 two-tailed) and control/OTXVP16 (U_OTXVP16_ = 15582.5, n(emb)_OTXVP16_ = 98, n_ctrl_ = 131, P<2.2e-16 two-tailed). (D) Distributions of cell numbers in the ANB and ASV after co-electroporation of Distal*Pitx*:lacZ and OTX fusions (yellow: control, red: enR fusion, blue: VP16 fusion; X-axis: cell numbers, Y-axis: proportions of embryos). Each experiment has been performed twice.

A functional study using morpholino antisense oligonucleotides in *Halocynthia roretzi* - another ascidian species - showed that the *Hr-Otx* knockdown strongly perturbs anterior neuroectoderm development, mostly because it is required for early specification events in the gastrula [49]. To avoid this early effect, we used targeted expression of dominant-negative and hyper-active versions of the *Ci*-OTX protein to interfere with its endogenous activity specifically after gastrulation. We thus engineered protein chimeras between the *Ci*-OTX homeodomain and the *Drosophila* engrailed repressor peptide or the VP16 *trans*-activation domain to create dominant-negative (OTX:EnR) or hyper-active (OTX:VP16) forms, respectively. We then used the *Ci-Six3 cis*-regulatory DNA to drive expression of these fusion proteins in a region that encompasses the ANB (Figure S1). These constructs were co-electroporated with the *Ci*-Distal-*Pitx* reporter plasmid, which contains the D1 enhancer with the two essential O1 and O2 K50-*Paired* binding sites [36], and the number of anterior neuroectodermal cells expressing the reporter gene was scored at the mid-tailbud stage (Figure 3). In control embryos expressing a *Ci-Six3*:Venus construct, an average of 2.78 anterior neuroectodermal cells per embryo activated the *Ci-Pitx* reporter construct, which can be accounted for by the mosaic incorporation of the transgene in the four ANB cells (Figure 3A and 3C). In contrast, targeted expression of *Ci*-OTX fusion proteins significantly altered *Ci-Pitx* reporter gene expression in the anterior neuroectoderm: the engrailed fusion inhibited ANB expression, while OTX:VP16 produced ectopic activation in surrounding neuroectodermal cells (Figure 3B–3D). Notably, OTX:VP16 also boosts expression of the ab dimer construct, and is not sufficient to induce overexpression when coelectroporated with one dimer construct bearing one O mutation (data not shown). This indicates that OTX:VP16 indeed binds to the GATTA binding sites. These observations strongly suggest that *Ci*-OTX trans-activating inputs are required for D1 enhancer activity in the anterior neuroectoderm. In addition, widespread expression of *Ci*-*Otx* in the anterior neuroectoderm contributes to the broad D1 trans-activation potential that encompasses the ANB, anterior epidermis and anterior sensory vesicle and is probably defined in D1 by the conserved GATTA/TAATC duplicated sequences. We cannot exclude the possibility that endogenous *Ci-Pitx* maintains its own expression through the same GATTA/TAATC BS, which binds PITX as well as OTX proteins. However, *Otx* is the best candidate for the onset of D1 activity, which begins exactly at the same time as the onset of the endogenous *Ci-Pitx* expression.

### Tandems of OTX binding sites preferentially flank anterior neuroectodermal and ectodermal genes

Of the three different duplicated BS that we identified for the ANB expression domain of *Pitx* and that we suppose to be specific to this restricted area of the anterior neuroectoderm, we concentrated our effort only on the binding sites for OTX as these are the only ones assignable to a well-characterized transcription factor. The observation that the transcriptional response to the broadly expressed head field-selector gene *Otx* is mediated by duplicated GATTA motifs led us to investigate whether this regulatory architecture was overrepresented in candidate *Otx* target genes in early tailbud embryos. At this stage, *Ci-Otx* expression extends over a broad domain referred to as in the anterior neuroectoderm, which derives from the a-line blastomeres and encompasses the ANB as well as other specific neurectodermal territories such as the anterior sensory vesicle, palps, a-line epidermis and rostral trunk epidermal neurons (RTEN). Therefore, we reasoned that candidate Otx target genes could, in principle, be expressed in all or part of the anterior neurectoderm. Hence, we asked whether duplicated GATTA motifs –the candidate signature for Otx binding- were enriched in the conserved noncoding sequences flanking genes with conspicuous expression in the anterior neurectoderm.

To this end, we obtained whole mount *in situ* hybridization data for 1518 genes showing tissue-specific expression from the model organism database ANISEED (December 2007, http://crfb.univ-mrs.fr/aniseed, see also Protocol S1). From these, we selected genes that are expressed in the central nervous system (CNS) and the ANB and classified them into different territories according to their expression along the antero-posterior axis: following previous reports [49]–[51], the ascidian visceral ganglion and the nerve cord were considered as “posterior” CNS whereas the whole sensory vesicle, including the ANB, constitute the “anterior” nervous system. This lead to a detailed annotation of nervous system expression patterns for 258 genes (Table S2). From this list we retained only those 100 genes that are specifically expressed in the anterior and not the posterior parts of the CNS. Finally, we obtained annotations for additional genes expressed in tissues like muscle, epidermis or notochord, from the database ANISEED. This latter set of genes was used as negative controls, which allowed for background definition for further statistical analyses. In total, our set includes annotations for 904 genes.

We then aimed at studying the distribution of duplicated short DNA motifs around these 904 genes to find those that show a bias towards genes expressed in the anterior or posterior nervous system, muscle, epidermis or notochord. We concentrated on conserved non-coding elements (CNEs), as these have been shown to be enriched in developmental enhancers [52], [53]. To obtain these elements for the genome of *Ciona intestinalis*, we created a whole-genome alignment with *Ciona savignyi* [54] and removed aligned positions in transcribed regions from it. This results in 168306 CNEs with an average length of 143 bp.

Then, we searched for duplicate matches to all 512 possible pentamers within 125 bp of all CNEs in the *Ciona intestinalis* genome and subsequently calculated the number of tissue-specific neighboring genes associated to each duplicated conserved pentamer and tissue. The rationale for using consensus and not matrix-based searches was that all subclasses of homeodomain proteins have well characterized binding sites that resemble pentamer motifs without degenerate positions [25], [26]. For the window size parameter, we observed from our case study that the sites had to occur in duplicates with a maximum distance of about 125bp, which was the total length of the fragment between both OTX-sites in the 75bp spacer construct. The score we chose was inspired by [55]; it does not require a sequence background model. This “motif-tissue-score” is the negative logarithm of the binomial probability to obtain a certain number of annotated genes from a given tissue by chance and therefore reflects the association of individual pentamer motifs with specific tissues.

Our first observation was that a duplicated OTX (GATTA) motif within 125 basepairs appears among the motifs with the highest score in the anterior CNS region (Table S3). For instance, genes containing duplicated GATTA motifs within 125bp in their flanking conserved genomic DNA are more likely to be expressed in the anterior nervous system than in any of the other tissues used in this analysis, including the posterior CNS (26% versus 12% or less, Table 1).

**Table 1:** Antero-posterior distribution of enhancers with 2×GATTA tags.

Tissue	Genes in this category	Genes flanked by 2×GATTA/125 bp in a conserved non-coding alignment	Percentage
anterior nervous system (specific)	100	26	0.26 *
posterior nervous system (specific)	58	7	0.12 *
notochord	346	18	0.05
epidermis	523	26	0.04
muscle	143	4	0.02

The percentage of positive anterior nervous system and positive posterior nervous system genes flanked by two GATTAs are significantly different (P = 0.043) according to a Fisher Exact two-tailed test.

**Figure 4:**
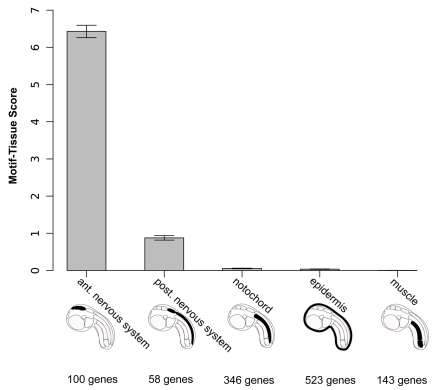
Motif-tissue scores for the motif 2×GATTA/125bp against genes expressed in various tissues. These territories are also visualized on schematic representation of an ascidian tailbud embryo. The number of genes is indicated for each category. To illustrate that changes in gene annotation are very unlikely to affect the overall ranking, we shuffled 10% of the gene-tissue assignments, repeated the procedure 100 times and plotted 95%-confidence intervals with error bars.

We then set out to assess the robustness of this analysis to variations of all three parameters: copy-number, window size and gene annotation. We varied the number of motif-duplicates from one to four and still obtained the highest motif-tissue scores in the anterior region with two copies. Increasing the window size from 25bp to 300bp did not change the scores to a large extent and the relative order between the anterior nervous system and other tissues always remained the same (Figure S3). The influence of errors in the manual annotation process was investigated by a simulation: we randomized 10% of all gene annotations and repeated this procedure 100 times. The 95% confidence intervals from these are small compared to the total differences between the tissues (Figure 4).

These results indicate that a biased distribution of GATTA motifs in CNEs supports the model of anterior ectodermal expression based on D1 enhancer analysis. We conclude that the presence of duplicated and conserved OTX binding sites in a cis-regulatory element is a signature for anterior neuroectoderm enhancer activity.

### Duplicated GATTA–motifs identify functional anterior ectoderm enhancers

**Figure 5:**
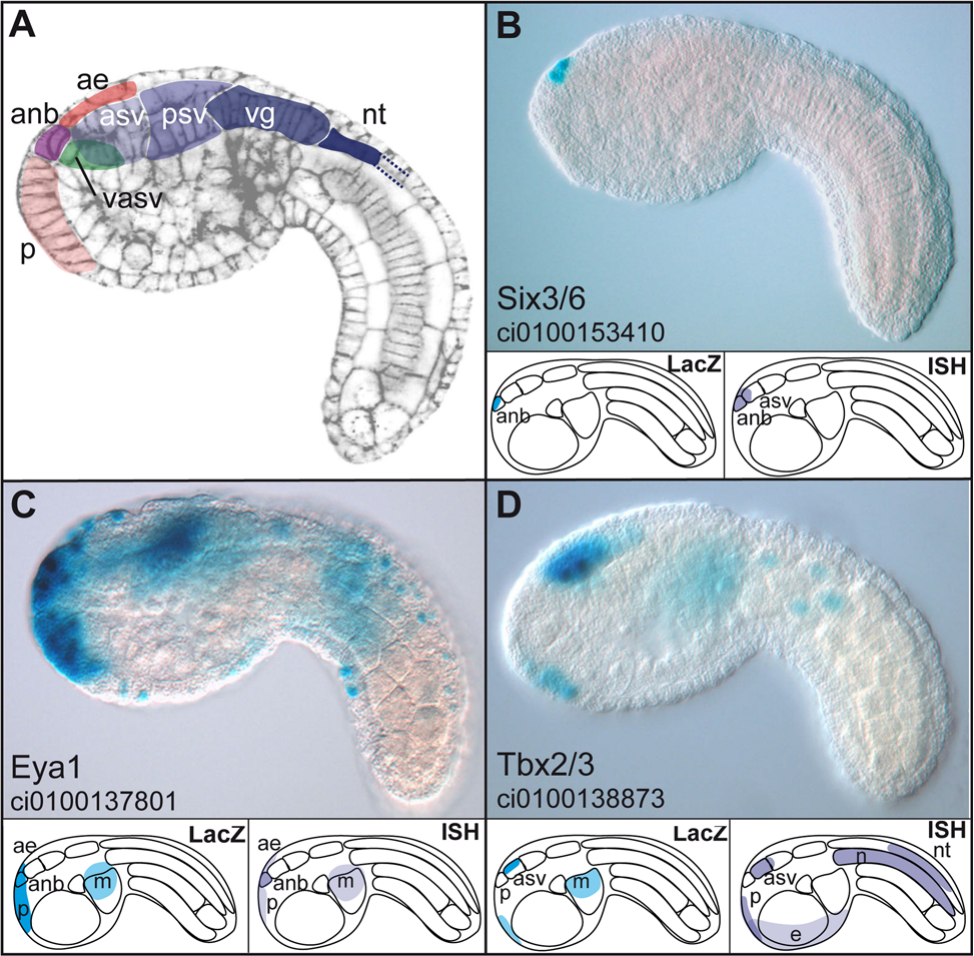
Enhancers with duplicated GATTA are active in the anterior region of the ascidian embryo. (A) Schematic representation of the main regions of gene expression in a mid-tailbud *Ciona intestinalis* embryo. Cell cortices are stained with Alexa-phalloidin (Christiaen et al. 2007). (B–D): expression domains of three enhancers, respectively from *Ci-Six3/6*, *Ci-Eya1* and *Ci-Tbx3* after electroporation and X-Gal staining at mid-tailbud stage. Lower panels show a schematic representation of the LacZ expression driven by the enhancer (left) and endogenous gene expression as assayed by *in situ* hybridization (ISH) (right). Enhancers can be subdivided into different classes following their expression domains: very restricted expression only in the ANB while the gene expression domain is slightly larger (Six3, (B)); broad anterior expression recapitulating more or less the endogenous expression pattern (Eya, (C)); only the most anterior expression domains are driven by the enhancer (Tbx3 (D)). anb: anterior neural boundary, asv: anterior sensory vesicle, psv: posterior sensory vesicle, vg: visceral ganglion, nt: neural tube, ae: anterior epidermis (or epineural epidermis), p: palps (precursors), m: mesenchyme, n: notochord. Lateral views, anterior to the left.

We then sought to test whether conserved sequences containing duplicated GATTA motifs act as enhancers in the anterior neuroectoderm. Out of all 53 CNEs with at least two conserved GATTAs in a 125 bp window that flank genes expressed in the anterior nervous system, we selected 30 CNEs. We succeeded in cloning 23 of them into a lacZ expression vector. After electroporation, we observed that ten of them are active enhancers in various domains of the anterior neuroectoderm derivatives, where *Otx* is expressed at the tailbud stage (Figure 5, Figure S2, and Table S4). The remaining non-coding regions were inactive or drove non-specific expression in the mesenchyme, as is often observed in electroporated ascidian embryos [56], [57]. This ratio of positive elements is high compared to a previously published enhancer screen of random DNA fragments (5 active enhancers out of 138 tested fragments) [57] and similar to a prediction based on binding site occurrences in *Drosophila* muscle founder cells (6 out of 12 tested elements) [58].

We were unable to identify additional motifs that would be predictive of enhancer activity in the anterior neurectoderm (Figure S4). However, additional motifs are required in natural enhancers, as we showed that pentamers of GATTA alone were unable to drive reporter gene activity in the Otx expression domain (data not shown). The diversity of expression patterns obtained with the ten active enhancers rather suggests that different transcription factors, each specific for a subdomain of the anterior neuroectoderm, might be implied in the activity of these elements.

Thus, while there might be additional motifs necessary for anterior neuroectoderm expression, this study shows the importance of the duplicated GATTA regulatory architecture as a predictive tag for the identification of anterior enhancers in chordates.

Could a signature based on GATTA-sites also be predictive in vertebrates? [52] reported that GATTA is over-represented in forebrain enhancers and used it as one of six motifs to predict forebrain enhancers in the mouse genome. We also found other overrepresented motifs in anteriorly expressed genes (see Table S3). Therefore, as determined experimentally with the D1 element, additional complexity must supplement the duplicated GATTA sites to achieve a cell-specific expression. Similar approaches performed in *Drosophila* and *Caenorhabditis* have identified several binding sites, which correspond to factors that specify a particular fate or behaviour in a combinatorial fashion, such as the myogenic factors [58],[59]. However, our study identifies for the first time a *cis*-regulatory signature that determines the transcriptional response to a “master” homeobox gene in a simple chordate and establishes a model for genome-wide predictions of tissue-specific enhancers.

## Materials and Methods

### Animals

Adult *Ciona intestinalis* were purchased at the Station de Biologie Marine de Roscoff (France) and maintained in artificial sea water at 15°C under constant illumination. Eggs and sperm were collected from dissected gonads and used in cross fertilizations. Electroporations, using 70 µg of DNA, and LacZ stainings were performed as previously described [36]. Embryo staging at 13°C were done according to [60], [61]. Images were taken on a Leica DMR microscope.

### Mutations in D1bcde

For the mutational analysis of the enhancer D1bcde (Figure 1), we omitted the first 16 bp (AAACGCGACGACCTCC) of D1abcde that were not conserved between *Ciona intestinalis* and *savignyi*. Each of the mutations was designed to perturb DNA-binding of the candidate *trans*-acting factors following various reports in the literature. Mutations were performed using the Stratagene QuickChange Kit. Seven new constructs called m0, m1, m2, m3, m4, m5/6, m7/8/9 were generated. After each electroporation, we observed LacZ expression in the tissues of the anterior neural boundary, anterior epidermis, ventro-anterior sensory vesicle and mesenchyme. We obtained a semi-quantitative estimation of the promoter activity by calculating the percentage of positive embryos.

### Artificial enhancers

Plasmids with artificial enhancers were designed by cloning inserts into the pCES2::lacZ vector that contains the basal *Ci-Fkh/FoxA* promoter [57]. Insert D1(ab) was generated by cloning two long complementary primers with XhoI/XbaI cohesive ends into pCES2. Inserts (abde), (abd), (ab)(ab-P^del^), (ab)(ab-O^mut^), (ab)(ab-T^mut^), (ab)(ab-G^mut^) were generated by cloning a second insert consisting of another couple of long complementary primers into the XbaI/BamHI site of D1(ab). The insert of D1(ab)×5 was designed *in silico*, synthetized by Genecust Europe (Luxembourg) and cloned into pCES2::LacZ between XhoI and BamHI. To obtain D1(ab)(ab), we cut out the first two parts of D1(ab)×5 with SalI/XhoI and ligated them into pCES2.

The spacer sequence between both (ab) parts of D1(ab)-xx-(ab) constructs was created *in silico* by avoiding all octamers bound by homeodomain factors from a large-scale DNA-protein binding assay [25]. We recursively added random nucleotides to an unbound sequence and backtracked if the new sequence contained an octamer with PBM enrichment score >0.3 from the UniProbe database [62]. These constructs, D1(ab)-xx-(ab) are also derived from D1(ab), but the insert was synthesized by GeneScript Corporation (Piscatway, NJ, USA). We amplified spacers of the appropriate length by PCR from the longer fragment and cloned them between the two duplicated (ab) fragment by restriction/ligation.

### OTX fusions

A pSix3:Venus plasmid was digested by BamHI/EcoRI to eliminate the Venus/YFP reporter.

#### VP16 fusion

the OTX_HD_ fragment was amplified by PCR from tailbud *Ciona* cDNA using OTX_HD_-F (CGGGATCCACAATGGTATACAGTTCGTCTAGAAAA) and OTX_HD_-R (AAACCATGGGTTGTTGCACTTGTTGGCGACA) oligos and digested by BamHI/NcoI. The VP16 domain was amplified with VP16-F (AAGATATCGACAAACCATGGTGCAGCTGGCACCACCGACCGATGTCAG) and VP16-R (AACAGCTGGAATTCTTAGATATCCCCACCGTACTCGTCAATTC) oligos, and digested by NcoI/EcoRI. Both resulting fragments were ligated into the linearized pSix3 driver to obtain the pSix3:OTX_HD_:VP16 construct.

#### EnR fusion

the OTX_HD_ fragment was amplified by PCR from tailbud cDNA using OTX_HD_-F (CGGGATCCACAATGGTATACAGTTCGTCTAGAAAA) and OTX_HD_-R (AAACCATGGGTTGTTGCACTTGTTGGCGACA) oligos. The enR repressor domain was amplified with enR-F (CTCGAGGCCCTGGAGGATCGC) and enR-R (CGAATTCTATACGTTCAGGTCCT) oligos. Both fragments were fused by additionnal rounds of PCR using oligos that overlap the 3′ part of OTX_HD_ and the 5′ part of enR (enR(OTX)F:TGTCGCCAACAAGTGCAACAACTCGAGGCCCTGGAGGATCGC, OTX_HD_(enR) R: GCGATCCTCCAGGGCCTCGAGTTGTTGCACTTGTTGGCGACA). The resulting product was digested by BamHI/EcoRI and ligated into into the digested pSix3 driver to obtain the pSix3: OTX_HD_:enR construct.

### Constructs for the enhancer screen

Plasmids containing non-coding elements were created with the Gateway Technology System (Invitrogen Carlsbad, CA, USA). We cloned an AttR3/AttR4 Gateway Cassette from [63] into the XhoI/XbaI-site of pCES2 and called the resulting construct AttR3R4-pCES2. Predicted fragments were first amplified by primers including part of the flanking AttB3/AttB4-sequences and then extended by a subsequent PCR to the full length sequences of AttB3/AttB4. These fragments were recombined with BP clonase into the P3/P4-donor Vector [63] and the resulting entry vectors recombined with LR clonase into AttR3R4-pCES2 producing expression vectors.

### In silico methods

Computational methods are described in Protocol S1. Programs that were used for whole-genome analyses are accessible at http://genome.ciona.cnrs-gif.fr/scripts/.

## Supporting Information

Figure S1 The pSix3 driver. (A) Vista plot of the *Ci-Six3/6* locus, showing conserved sequences between *Ciona intestinalis* and *Ciona savignyi*: exons in blue and non-coding sequences in pink. The pSix3 construct (blue line) encompasses the 2 kb upstream the initiator codon of the SIX3 protein. (B) *Ci-Six3/6* gene is expressed at the early neurula stage in the most anterior neural plate cell row (C) it continues to be expressed at the tailbud stage in the ANB and the anterior sensory vesicle. (D, E) The pSix3 construct drives reporter gene expression in a similar way to the endogenous gene at neurula and tailbud stages. It was used to drive expression of dominant-negative and hyper-active forms of the *Ci*-OTX protein in ANB precursor cells (Figure 3).
(3.20 MB PDF)

Figure S2 Images of LacZ-stained embryos and genomic context of the CNEs. Pictures of mid-tailbud stage embryos in side view electroporated with enhancer:pCES2:lacZ constructs and position of the enhancer on the *Ciona* genome. When more than one GATTA-rich enhancer has been found in a locus, we chose to clone one or two of them, according to the following criteria: the conservation on the vista plot (higher conservation and unique peak were preferentially chosen), the absolute number of conserved GATTA, the proximity and relative position to the transcription start (intronic and 5′ were preferred to 3′). Conserved regions with duplicated GATTAs on the first track. Positive enhancers are represented in blue boxes, negative regions in empty blue boxes, other regions in grey. The number of conserved GATTAs in the region is indicated above the boxes. When available, the best matching human gene model (BLAST), the JGI1 gene model number and VISTA conservation are shown below. (A, B) Six3/6: expression of the lacZ reporter in the ANB. (C, D) Eya1: expression in the ANB, anterior head and palp epidermis, mesenchyme, and weak expression in the tail epidermis. (E, F) Tbx3: expression in the dorsal part of the ASV and weaker in the palp epidermis. (G, H) FoxF: expression in the head epidermis surrounding the ASV. (I, J) Ets1: expression in the dorsal ASV, palps epidermis and tail tip epidermis less frequently. (K,L) Efnb3: broad expression in the anterior head epidermis. (M, N) Zswim: expression in the ASV, the PSV and weaker expression in the notochord and the tail epidermis. (O, P) Otx: expression in the most posterior part of the ASV and in the PSV. (Q, R) ci0100154565: expression in the ANB, the posterior sensory vesicle and weak in the mesenchyme. (S, T) Hes1: expression in the ASV and broad expression in the mesenchyme and tail muscles and epidermis. Endogenous expressions can be seen in Table S2.
(8.16 MB PDF)

Figure S3 Motif-tissue scores of the 2×GATTA *cis*-regulatory signature depending on window size. Window size between two GATTA motifs has been varied from 25 to 300 bps. It can be seen that the window size parameter does not to a large extent influence the results and never changes the order of the tissues; the anterior nervous system is always the highest ranking tissue. Procedures are described in Protocol S1.
(0.50 MB PDF)

Figure S4 Motif occurrences in predicted enhancers. The number of putative binding site motifs from Figure 1 (T and G motifs) and the two topscoring motifs from Table S3 are represented by colors according to the legend on the right. The name of the constructs refer to the regions electroporated and described in Table S4. (−) indicates inactive and (+) indicates active construct (grouped in the red box). All constructs contain at least two GATTA motifs as this was the main criteria for their identification. As can be seen from this diagram, no motif occurs preferentially in the positive constructs.
(0.24 MB PDF)

Table S1 Timing of D1bcde-driven reporter gene expression.
(0.40 MB PDF)

Table S2 Annotation of expression patterns for 258 genes at early-/mid-tailbud stages.
(1.40 MB PDF)

Table S3 Motif-Tissue-Scores of the ten highest-scoring motif duplicates in the anterior and posterior nervous system.
(0.30 MB PDF)

Table S4 Overview of enhancer screen CNEs and stained territories after electroporation.
(0.43 MB PDF)

Protocol S1 *In silico* protocols.
(0.03 MB PDF)
