# Supplementary material for: A cis-Regulatory Signature for Chordate Anterior Neuroectodermal Genes
Source: PLoS Genet. 2010 Apr 15;6(4):e1000912. doi: 10.1371/journal.pgen.1000912 (PMC2855326; doi:10.1371/journal.pgen.1000912)
Supplement: Figure S1 — The pSix3 driver. (A) Vista plot of the Ci-Six3/6 locus, showing conserved sequences between Ciona intestinalis and Ciona savignyi: exons in blue and non-coding sequences in pink. The pSix3 construct (blue line) encompasses the 2 kb upstream the initiator codon of the SIX3 protein. (B) Ci-Six3/6 gene is expressed at the early neurula stage in the most anterior neural plate cell row (C) it continues to be expressed at the tailbud stage in the ANB and the anterior sensory vesicle. (D, E) The pSix3 construct drives reporter gene expression in a similar way to the endogenous gene at neurula and tailbud stages. It was used to drive expression of dominant-negative and hyper-active forms of the Ci-OTX protein in ANB precursor cells (Figure 3). (3.20 MB PDF) [file pgen.1000912.s001.pdf]

Figure S1: The pSix3 driver.

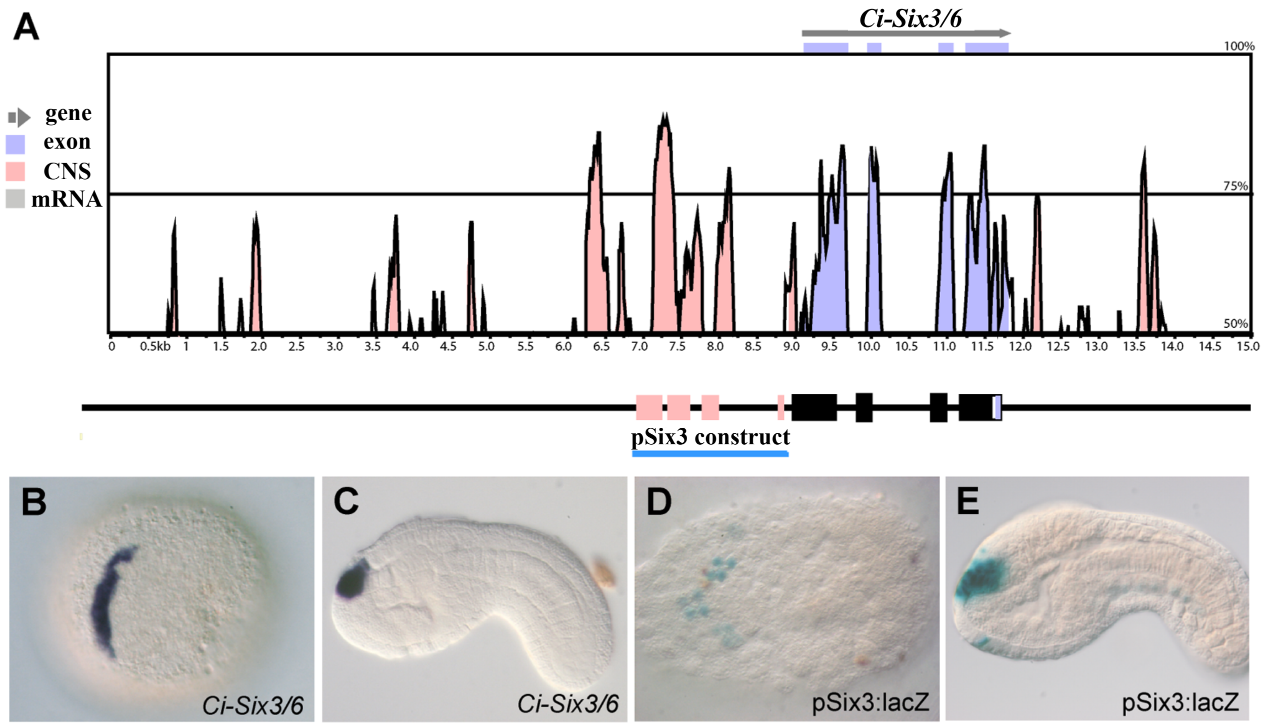

Figure S1: The pSix3 driver.

(A) Vista plot of the *Ci-Six3/6* locus, showing conserved sequences between *Ciona intestinalis* and *Ciona savignyi* : exons in blue and non-coding sequences in pink. The pSix3 construct (blue line) encompasses the 2 kb upstream of the initiator codon of the SIX3 protein. (B) *Ci-Six3/6* gene is expressed at the early neurula stage in the most anterior neural plate cell row (C) it continues to be expressed at the tailbud stage in the ANB and the anterior sensory vesicle. (D, E) The pSix3 construct drives reporter gene expression in a similar way to the endogenous gene at neurula and tailbud stages. It was used to drive expression of dominant-negative and hyper-active forms of the *Ci*-OTX protein in ANB precursor cells (Figure 3).
