# Supplementary material for: A cis-Regulatory Signature for Chordate Anterior Neuroectodermal Genes
Source: PLoS Genet. 2010 Apr 15;6(4):e1000912. doi: 10.1371/journal.pgen.1000912 (PMC2855326; doi:10.1371/journal.pgen.1000912)
Supplement: Figure S2 — Images of LacZ-stained embryos and genomic context of the CNEs. Pictures of mid-tailbud stage embryos in side view electroporated with enhancer:pCES2:lacZ constructs and position of the enhancer on the Ciona genome. When more than one GATTA-rich enhancer has been found in a locus, we chose to clone one or two of them, according to the following criteria: the conservation on the vista plot (higher conservation and unique peak were preferentially chosen), the absolute number of conserved GATTA, the proximity and relative position to the transcription start (intronic and 5′ were preferred to 3′). Conserved regions with duplicated GATTAs on the first track. Positive enhancers are represented in blue boxes, negative regions in empty blue boxes, other regions in grey. The number of conserved GATTAs in the region is indicated above the boxes. When available, the best matching human gene model (BLAST), the JGI1 gene model number and VISTA conservation are shown below. (A, B) Six3/6: expression of the lacZ reporter in the ANB. (C, D) Eya1: expression in the ANB, anterior head and palp epidermis, mesenchyme, and weak expression in the tail epidermis. (E, F) Tbx3: expression in the dorsal part of the ASV and weaker in the palp epidermis. (G, H) FoxF: expression in the head epidermis surrounding the ASV. (I, J) Ets1: expression in the dorsal ASV, palps epidermis and tail tip epidermis less frequently. (K,L) Efnb3: broad expression in the anterior head epidermis. (M, N) Zswim: expression in the ASV, the PSV and weaker expression in the notochord and the tail epidermis. (O, P) Otx: expression in the most posterior part of the ASV and in the PSV. (Q, R) ci0100154565: expression in the ANB, the posterior sensory vesicle and weak in the mesenchyme. (S, T) Hes1: expression in the ASV and broad expression in the mesenchyme and tail muscles and epidermis. Endogenous expressions can be seen in Table S2. (8.16 MB PDF) [file pgen.1000912.s002.pdf]

Figure S2: Enhancer screen results.

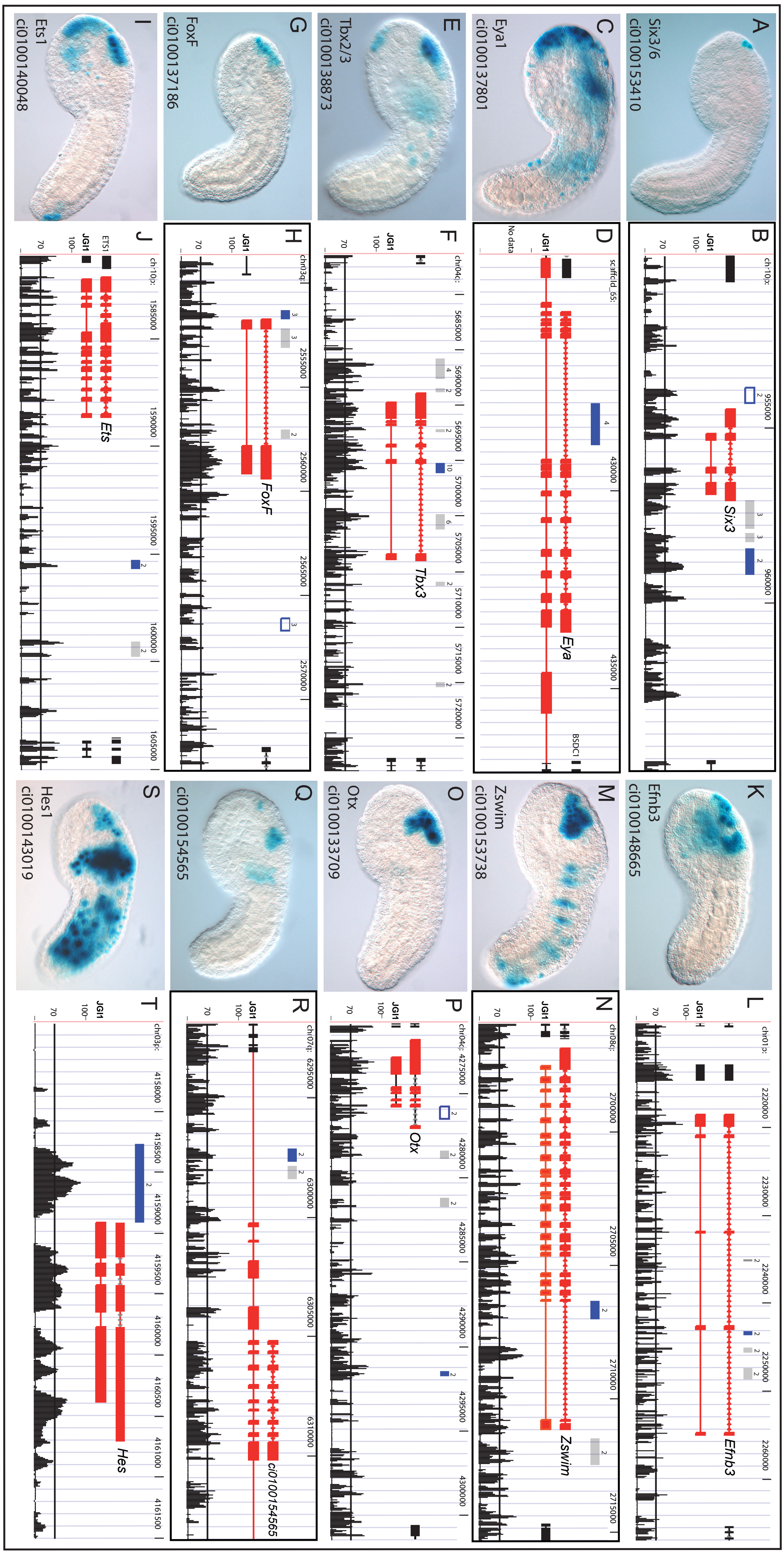

Figure S2: Enhancer screen results.

Pictures of mid-tailbud stage embryos in side view electroporated with enhancer:pCES2:lacZ constructs and position of the enhancer on the *Ciona* genome. When more than one GATTA-rich enhancer has been found in a locus, we chose to clone one or two of them, according to the following criteria: the conservation on the vista plot (higher conservation and unique peak were preferentially chosen), the absolute number of conserved GATTA, the proximity and relative position to the transcription start (intronic and 5' were preferred to 3'). Conserved regions with duplicated GATTAs on the first track. Positive enhancers are represented in blue boxes, negative regions in empty blue boxes, other regions in grey. The number of conserved GATTAs in the region is indicated above the boxes. When available, the best matching human gene model (BLAST), the JGI1 gene model number and VISTA conservation are shown below.

(A, B) Six3/6: expression of the lacZ reporter in the ANB. (C, D) Eya1: expression in the ANB, anterior head and palp epidermis, mesenchyme, and weak expression in the tail epidermis. (E, F) Tbx3: expression in the dorsal part of the ASV and weaker in the palp epidermis. (G, H) FoxF: expression in the head epidermis surrounding the ASV. (I, J) Ets1 : expression in the dorsal ASV, palps epidermis and tail tip epidermis less frequently. (K,L) Efnb3: broad expression in the anterior head epidermis. (M, N) Zswim: expression in the ASV, the PSV and weaker expression in the notochord and the tail epidermis. (O, P) Otx: expression in the most posterior part of the ASV and in the PSV. (Q, R) ci0100154565: expression in the ANB, the posterior sensory vesicle and weak in the mesenchyme. (S, T) Hes1: expression in the ASV and broad expression in the mesenchyme and tail muscles and epidermis. Endogenous expressions can be seen in Table S2.
