# Supplementary material for: A cis-Regulatory Signature for Chordate Anterior Neuroectodermal Genes
Source: PLoS Genet. 2010 Apr 15;6(4):e1000912. doi: 10.1371/journal.pgen.1000912 (PMC2855326; doi:10.1371/journal.pgen.1000912)
Supplement: Figure S3 — Motif-tissue scores of the 2×GATTA cis-regulatory signature depending on window size. Window size between two GATTA motifs has been varied from 25 to 300 bps. It can be seen that the window size parameter does not to a large extent influence the results and never changes the order of the tissues; the anterior nervous system is always the highest ranking tissue. Procedures are described in Protocol S1. (0.50 MB PDF) [file pgen.1000912.s003.pdf]

Figure S3 : Motif-tissue scores of the 2xGATTA *cis*-regulatory signature depending on window size.

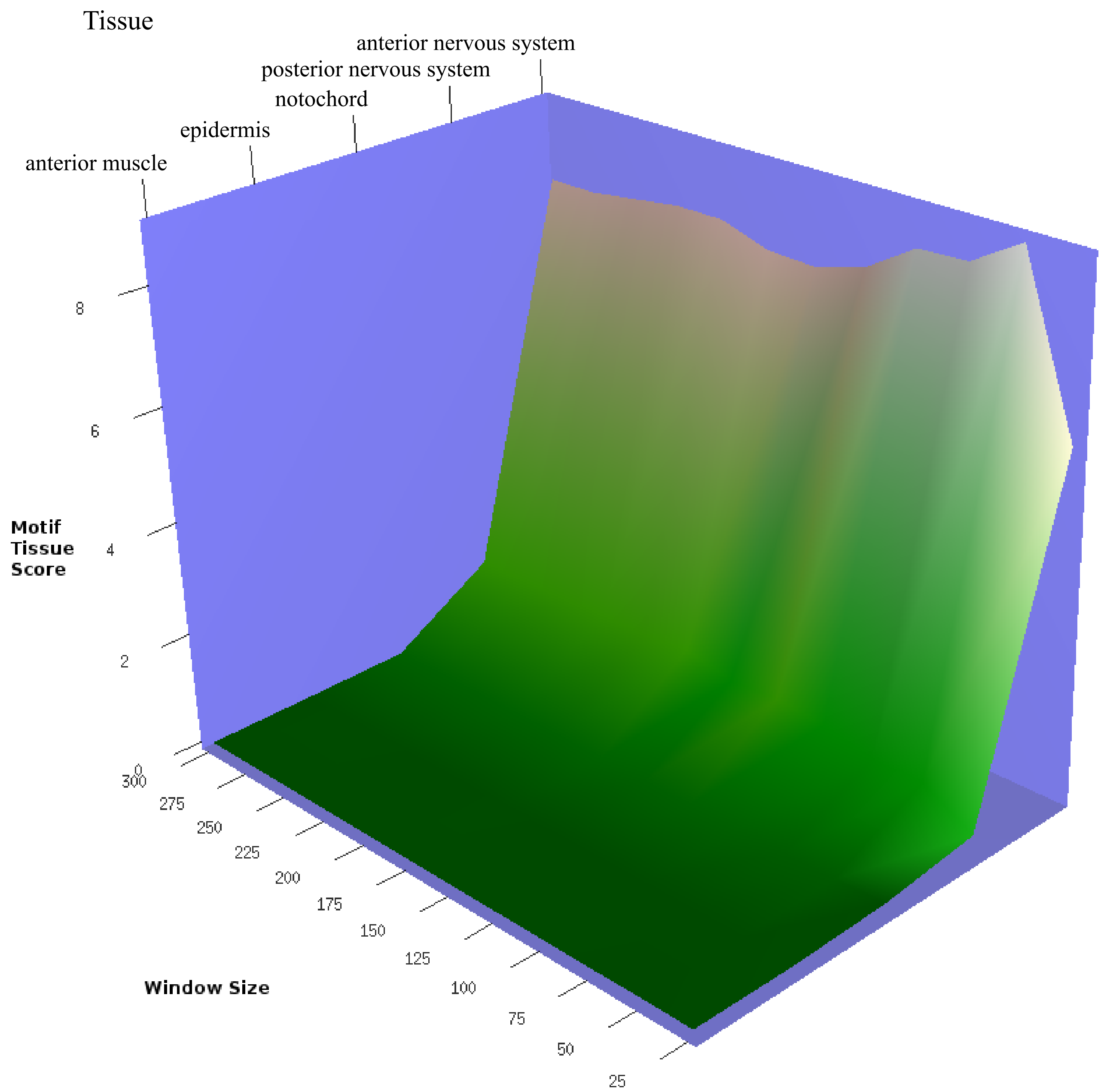

Figure S3: Motif-tissue scores of the 2xGATTA *cis*-regulatory signature depending on window size. Window size between two GATTA motifs has been varied from 25 to 300 bps. It can be seen that the window size parameter does not to a large extent influence the results and never changes the order of the tissues; the anterior nervous system is always the highest ranking tissue. Procedures are described in Protocol S1.
