# Supplementary material for: A cis-Regulatory Signature for Chordate Anterior Neuroectodermal Genes
Source: PLoS Genet. 2010 Apr 15;6(4):e1000912. doi: 10.1371/journal.pgen.1000912 (PMC2855326; doi:10.1371/journal.pgen.1000912)
Supplement: Figure S4 — Motif occurrences in predicted enhancers. The number of putative binding site motifs from Figure 1 (T and G motifs) and the two topscoring motifs from Table S3 are represented by colors according to the legend on the right. The name of the constructs refer to the regions electroporated and described in Table S4. (−) indicates inactive and (+) indicates active construct (grouped in the red box). All constructs contain at least two GATTA motifs as this was the main criteria for their identification. As can be seen from this diagram, no motif occurs preferentially in the positive constructs. (0.24 MB PDF) [file pgen.1000912.s004.pdf]

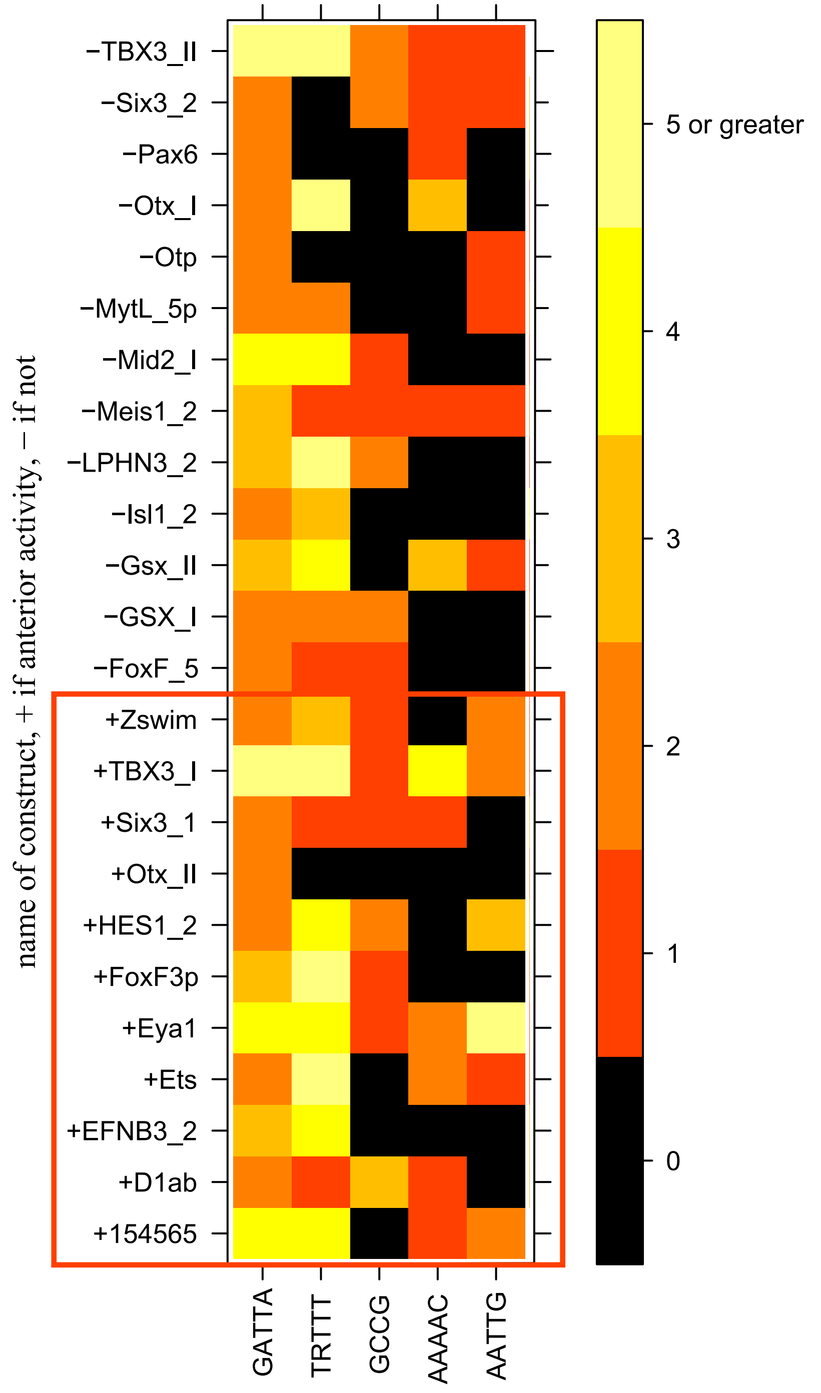

Figure S4 Motif occurences in predicted enhancers.

The number of putative binding site motifs from Figure 1 (T and G motifs) and the two top-scoring motifs from Table S3 are represented by colors according to the legend on the right. The name of the constructs refer to the regions electroporated and described in Table S4. (-) indicates inactive and (+) indicates active construct (grouped in the red box). All constructs contain at least two GATTA motifs as this was the main criteria for their identification. As can be seen from this diagram, no motif occurs preferentially in the positive constructs.
