# Supplementary material for: A cis-Regulatory Signature for Chordate Anterior Neuroectodermal Genes
Source: PLoS Genet. 2010 Apr 15;6(4):e1000912. doi: 10.1371/journal.pgen.1000912 (PMC2855326; doi:10.1371/journal.pgen.1000912)
Supplement: Table S1 — Timing of D1bcde-driven reporter gene expression. (0.40 MB PDF) [file pgen.1000912.s005.pdf]

Table S1: Timing of D1bcde-driven reporter gene expression

Stage (dev. time*)	Staining method	Total embryos	Stained embryos (N)	ANB %‡ (n)	ae, vasv # %‡ (n)	Mesenchyme %‡ (n)
**iTB** (16.5hpf)	X-gal	119	71 (59.7%)	**7.0%** (5)	5.6% (4)	97.2% (69)
	ISH	126	83 (65.9%)	**55.4%** (46)	36.1% (30)	75.9% (63)
eTB (17.5hpf)	X-gal	73	43 (58.9%)	**60.5%** (26)	27.9% (12)	90.7% (39)
	ISH	175	119 (68.0%)	67.2% (80)	52.9% (63)	88.2% (105)
mTB (18.5hpf)	X-gal	162	109 (67.3%)	78.0% (85)	48.6 (53)	91.7% (100)
	ISH	n.d.	n.d.	n.d.	n.d.	n.d.

* The development time indicated is for embryos actually raised at 13°C.

‡ The percentage of embryos showing expression in the indicated tissues was calculated referring to the number of stained embryos (note that the proportion of stained embryos is about the same in all conditions, but slightly increased when using *in situ* hybridization –ISH- because this method is more sensitive than X-gal staining). This calculation method makes the proportion of mesenchyme expressing embryos to drop while the proportion of ANB expressing embryos increases because some embryos show ANB staining but no mesenchyme staining. However, when calculated over the total of embryos, the proportion of mesenchyme expressing embryos remains about the same in all conditions (50-60%).

# the ae, vasv category includes embryos showing staining in either the anterior epidermis (ae) and/or the ventro anterior sensory vesicle (vasv).

iTB: initial tailbud, eTB: early tailbud, mTB: mid-tailbud, ISH: *in situ* hybridization, ANB: anterior neural boundary.
