# Supplementary material for: A cis-Regulatory Signature for Chordate Anterior Neuroectodermal Genes
Source: PLoS Genet. 2010 Apr 15;6(4):e1000912. doi: 10.1371/journal.pgen.1000912 (PMC2855326; doi:10.1371/journal.pgen.1000912)
Supplement: Table S3 — Motif-Tissue-Scores of the ten highest-scoring motif duplicates in the anterior and posterior nervous system. (0.30 MB PDF) [file pgen.1000912.s007.pdf]

Table S3: Motif-Tissue-Scores of the ten highest-scoring motif duplicates in the anterior and posterior nervous system.

Duplicated Motif within 125bp	Motif-Tissue score in anterior nervous system genes	Motif-Tissue score in posterior nervous system genes
2xAAAAC	9.73	0.35
2xAATTG	9.27	0.69
2xGATTA	8.27	0.84
2xAAACA	7.77	1.38
2xATTAG	7.01	0.31
2xAAATC	6.81	0.12
2xATTAA	6.76	2.01
2xAATTA	6.73	1.71
2xAAAGC	6.59	1.15
2xAACAA	6.44	2.81
