# Supplementary material for: A cis-Regulatory Signature for Chordate Anterior Neuroectodermal Genes
Source: PLoS Genet. 2010 Apr 15;6(4):e1000912. doi: 10.1371/journal.pgen.1000912 (PMC2855326; doi:10.1371/journal.pgen.1000912)
Supplement: Table S4 — Overview of enhancer screen CNEs and stained territories after electroporation. (0.43 MB PDF) [file pgen.1000912.s008.pdf]

gene name	gene model	position/gene	size(pb)	position/genome (assembly 2005)	expression domains
D1 (pitx)	ci0100147848	5'	220	Scaffold_63:217,283-217,498	ANB
Eya1	ci0100137801	intron	1239	scaffold_65:427,730-428,968	ANB, anterior head epidermis, palps, mesenchyme
Six3 (I)	ci0100153410	5'	161	chr10p:958,511-958,671	ANB, anterior head epidermis, palps, mesenchyme
Efnb3	ci0100148665	intron	698	chr01p:2242681-2244081	anterior head epidermis
Hes1	ci0100143019	5'	929	chr03p:4157771-4159410	ASV, tail muscles, mesenchyme
Tbx2/3	ci0100138873	intron	1036	chr04q:5694673-5696662	ASV, anterior head epidermis
ci0100154565	ci0100154565	5'	915	chr07q:6296611-6298207	ASV, anterior head epidermis
FoxF_3'	ci0100137186	3'	613	chr03q:2550777-2552248	ASV, head epidermis, mesenchyme
Otx (II)	ci0100133709	5'	402	chr04q:4291433-4291769	ASV, PSV
Ets	ci0100140048	5'	743	chr10p:1,599,065-1,599,760	ASV, palps, tail tip epid., mesench.(weak)
Zswim	ci0100153738	5'	675	chr08q:2711850-2712450	ASV, palps, tail tip epid, mesench., notocord (weak)
Onecut	ci0100139609	intron	738	chr06q:719674-720358	-
FoxF_5'	ci0100137186	5'	681	chr03q:2565610-2567201	-
Otx (I)	ci0100133709	intron	740	chr04q:4276015-4276514	-
Six3 (II)	ci0100153410	3'	611	chr10p:953564-954601	-
Otp	ci0100132453	5'	360	chr14q:2222502-2223394	-
Gsx (I)	ci0100151030	5'	277	chr02q:5891182-5892355	-
Gsx (II)	ci0100151030	5'	321	chr02q:5890790-5891043	-
Isl1	ci0100134923	intron	865	scaffold_95:332634-334380	-
Mid2	ci0100137572	intron	384	chr02q:4278147-4279423	-
Meis	ci0100130253	intron	502	chr10q:2080805-2082233	-
Lphn3	ci0100150579	intron	454	chr05q:6140041-6141425	-
MytL	ci0100131573	5'	699	chr01q:1546517-1547123	-
Pax6	ci0100144072	5'	221	chr09q:3329200-3329372	-

Table S4: Enhancer screen

List of regions cloned for the enhancer screen. Gene name indicates the most closely located gene to the tested element, and the corresponding gene model is indicated in the next column. The type of location of the element relatively to the gene is indicated in the third column. The size of the tested element is reported in the fourth column. The position of the element in the Ciona intestinalis genome (2005 JGI assembly) is reported in the fifth column and expression domain of each enhancer in the last column. Note that all enhancers without expression or with weak expression in the mesenchyme were indicated as “negative”.
