# Supplementary material for: A cis-Regulatory Signature for Chordate Anterior Neuroectodermal Genes
Source: PLoS Genet. 2010 Apr 15;6(4):e1000912. doi: 10.1371/journal.pgen.1000912 (PMC2855326; doi:10.1371/journal.pgen.1000912)
Supplement: Protocol S1 — In silico protocols. (0.03 MB PDF) [file pgen.1000912.s009.pdf]

Supporting protocol : *In silico* protocols.

Annotating the expression pattern of genes in the nervous system.

We used a December 2007 version (provided by Fabrice Daian and Patrick Lemaire) of the database Aniseed (http://crfb.univ-mrs.fr/aniseed/) which is based to a large extent on images obtained from several large-scale whole-mount *in-situ* hybridization screens [1,2,3,4]. We only selected genes with a JGI Version 1 gene identifier that are expressed at early- or mid-tailbud stages (2396 genes) and removed all genes with the expression annotation "whole embryo" or "not expressed" at any of these two stages. This resulted in 1518 genes.

As control gene sets, we selected genes expressed in the territories "primary muscle", "epidermis" and "notochord", using the annotation in Aniseed (see Table 1 for the total number of genes in these classes). *In situ* images are more difficult to annotate for nervous system sub-structures than for larger territories like muscle tissue or the epidermis. To improve the existing annotation in Aniseed, we copied the images from the Aniseed website and reannotated them manually using a simple web interface (http://genome.ciona.cnrs-gif.fr/scripts/insituFlash/insituFlashInit.cgi). The result of the manual annotation is a list that assigns each gene to one or several of the classes "palps", "stomodeum", "tip of anterior sensory vesicle", "anterior sensory vesicle", "posterior sensory vesicle", "visceral ganglion" and "nerve cord", again dropping images of embryos with a semi-ubiquitous or weak expression. We also added 31 genes based on data from [5] and two genes from [6]. The resulting gene-annotation list is available as Table S2.

A comparison between different territories can be confused by genes that are expressed in several domains at the same time. For example, we noted that many genes in the anterior nervous system are also coexpressed in the posterior part, mostly in the visceral ganglion (see Table S2), and therefore removed from the “anterior nervous system” class genes which are expressed both in the posterior nervous system (visceral ganglion, nerve cord) and the anterior nervous system (stomodeum, sensory vesicle). Table 1 summarizes the number of genes in these two categories and the respective share of genes flanked by 2xGATTA.

Searching for genes flanked by a duplicated pentamer contained in a conserved noncoding region.

To find genes where conserved flanking elements contain a combination of pentamers, we aligned the two repeatmasked genomes of *Ciona intestinalis* 2.0 and *Ciona savignyi* 2.0 using the UCSC BlastZ/Chain/Net/MultiZ pipeline as described in [7]. Whereas BlastZ might be less sensitive than the Vista-Pipeline [8], we chose BlastZ because we are mainly interested in well-conserved sequences and

because its integration with the UCSC alignment pipeline. Moreover, the Vista alignment of the genome in its latest version is completely lacking some loci, noteably the scaffold where PITX is located. The BlastZ alignment process is documented in [6] and also on http://genomewiki.ucsc.edu/index.php/Whole_genome_alignment_howto. The resulting alignments can be explored and downloaded from http://genome.ciona.cnrs-gif.fr. To retain only non-coding sequences for further analysis, we removed all basepairs that overlap exons or UTRs of Ensembl Release 44 gene models. These non-coding sequence alignments were subsequently annotated with their closest flanking JGI Version1 gene model ID to link them to *in-situ* annotations. The resulting alignment blocks cover 25.5 Mbp of the genome, organized in 168306 blocks that contain at least one stretch of five conserved nucleotides. The average length of these alignments on the *Ciona intestinalis* genome is 143 bp, the average number of completely conserved basepairs in them is 102bp.

As for the motifs to search in these alignments, we chose an exhaustive non-degenerate search of pentamers. This simple motif model has the advantage of reducing the overfitting problem inherent in all motif discovery approaches [9]. There are 512 pentamers corresponding to all possible combinations of five nucleotides. We searched the annotated consensus sequences for all possible 512 pentamers and kept only those where two identical pentamers occur within a certain distance and output the genes closest to these alignments. We set the default maximum distance from the first to the last motif to 125 bp as this corresponds to the experimental results.

Our program (which is also accessible as an interactive web interface at http://genome.ciona.cnrs-gif.fr/scripts/cionator2/wordSearchForm.cgi) can search the non-coding alignment blocks for a given number and combinations of pentamers within a certain distance. The program outputs genes that are flanked by these matches. To assign a P-Value to a given enrichment, we calculate the binomial probability as in [10] but adapted it to a gene-based annotation. We call the logarithm of this p-Value the "motif-tissue score"; it reflects how well matches of a motif flank genes expressed in a tissue.

Obtaining a motif - tissue score from the overlap between predicted and true positive genes and influence of different parameters.

To illustrate the motif-tissue score, we give an example of a search performed with the motif GATTA, against the tissue "anterior nervous system". The foreground in this case is the population of genes specifically expressed in the "anterior nervous system" (100 genes), while all genes annotated as "anterior nervous system", "posterior nervous system", "muscle", "epidermis" or "notochord" represent the background (904 genes).

Of the 904 genes, 100 genes are expressed in the anterior nervous system. In total, our set includes annotations for 904 genes. The probability to obtain a gene expressed in the "anterior nervous system"

without the knowledge of any motif is 11%. There are 68 matches to 2xGATTA/125bp in the genome; 26 of these (38%) are located in the anterior nervous system. Thus, using the GATTA-motif and testing many enhancers, one should obtain a four-fold enrichment of anterior nervous system enhancers. Using the binomial probability, we can calculate the p-Value, how probable it is to obtain 26 or more genes with anterior expression if the motif 2xGATTA had no influence on the result. The binomial probability to obtain 26 white balls or more when drawing 68 balls from an urn with 11% white balls is 5.42e-09. Taking the -log10 of this, we obtain the motif-tissue-score 8.26. This is very similar to the group specificity score of [9,11,12] but using the binomial probability instead of the hypergeometric one to simplify calculations, as in [10].

As can be seen from the example, the score is calculated as follows: Given a motif **m**, a list of **f** genes in the foreground and a list of **b** genes in the background, with **t** genes that are flanked (predicted) by a motif **m** and **x** genes that are flanked by motif m and are also in the foreground, we calculate the binomial probability to obtain **x** or more foreground-genes when randomly drawing **t** times, given that the probability to draw a foreground gene is the ratio of **f/b**.

We know from the D1bcde-mutations and artificial enhancer experiments that the essential motifs for activity in the anterior neurectoderm need to be present in two copies. Pentamers fit well the binding properties of *bicoid*-like proteins. Therefore, we limit our search to all duplicated pentamers. There are 1024 different pentamers AAAAA, AAAAT...etc... to TTTTT, removing reverse complements leaves 512 different pentamers. As we are testing 512 different hypotheses at the same time, the minimal p-Value is not 0.01 but 0.01 divided by the number of hypotheses (Bonferroni-correction), leading to a minimal motif score of 4.7 to be significant. Calculating this score on different tissues and on 10%-shuffled gene sets leads to the different diagrams that are part of Figure 4. To illustrate that the window size parameters does not have a large influence on the results and that the most important parameter is indeed the list of genes expressed in a given tissue, we have plotted motif-tissues scores for the motif 2xGATTA, different window sizes and tissues in Figure S3.

Other motifs with high motif-tissue scores

Our ranking also identified other motifs that are associated with anterior expression (See Table S2): The first is AAAAC which is also found twice in the minimal *Ci-pitx* enhancer but its mutation in the artificial enhancer lead to a drastic reduction but not a complete abolishment of the expression of the construct with one mutated T site (D1(ab)(ab-T^{mut}) of Figure 2D). We are only interested in essential motifs, those that completely abolish expression when mutated, therefore we did not further pursue the analysis of this motif.

The second motif is AATTG, which is found in the 3'-part of D1 enhancer and conserved with *Ciona savignyi*. However, it seems to be dispensable for expression in the anterior neural boundary because it

is present in a part of D1 that can be removed, absent from D1abbce. It represents a putative binding site for the transcription factor HMX1/2/3. This factor is conserved in many bilaterians and its expression pattern suggests an ancestral function in rostral development [13,14].

Impact of annotation errors

Annotation of in-situ patterns is a manual process and errors or omissions might influence the result. We tried to illustrate the impact of changes in it by a randomization experiment. We replaced 10% of the annotated genes by random genes out of the 1518 ones for which images are available, calculate the motif-tissue score for 2xGATTA and repeat this procedure 100 times. The results are shown in Figure 4 and illustrate that the absolute value of the top motifs might be indeed sensitive to annotation error but that a lower enrichment in the posterior parts than in the anterior parts of the nervous system is found in all trials.

References

1. Satou Y, Takatori N, Yamada L, Mochizuki Y, Hamaguchi M, et al. (2001) Gene expression profiles in Ciona intestinalis tailbud embryos. Development 128: 2893-2904.
2. Mochizuki Y, Satou Y, Satoh N (2003) Large-scale characterization of genes specific to the larval nervous system in the ascidian Ciona intestinalis. Genesis 36: 62-71.
3. Imai KS, Hino K, Yagi K, Satoh N, Satou Y (2004) Gene expression profiles of transcription factors and signaling molecules in the ascidian embryo: towards a comprehensive understanding of gene networks. Development 131: 4047-4058.
4. Satou Y, Kawashima T, Shoguchi E, Nakayama A, Satoh N (2005) An integrated database of the ascidian, Ciona intestinalis: towards functional genomics. Zoolog Sci 22: 837-843.
5. Ikuta T, Saiga H (2007) Dynamic change in the expression of developmental genes in the ascidian central nervous system: revisit to the tripartite model and the origin of the midbrain-hindbrain boundary region. Dev Biol 312: 631-643.
6. Auger H, Lamy C, Haeussler M, Khoueiry P, Lemaire P, et al. (2009) Similar regulatory logic in Ciona intestinalis for two Wnt pathway modulators, ROR and SFRP-1/5. Dev Biol.
7. Kent WJ, Baertsch R, Hinrichs A, Miller W, Haussler D (2003) Evolution's cauldron: duplication, deletion, and rearrangement in the mouse and human genomes. Proc Natl Acad Sci U S A 100: 11484-11489.
8. Visel A, Minovitsky S, Dubchak I, Pennacchio LA (2007) VISTA Enhancer Browser--a database of tissue-specific human enhancers. Nucleic Acids Res 35: D88-92.
9. MacIsaac KD, Fraenkel E (2006) Practical strategies for discovering regulatory DNA sequence motifs. PLoS Comput Biol 2: e36.
10. Xie X, Lu J, Kulbokas EJ, Golub T, Mootha V, et al. (2005) Systematic discovery of regulatory motifs in human promoters and 3[prime] UTRs by comparison of several mammals. Nature aop.
11. Hughes JD, Estep PW, Tavazoie S, Church GM (2000) Computational identification of cis-regulatory elements associated with groups of functionally related genes in Saccharomyces cerevisiae. J Mol Biol 296: 1205-1214.
12. Yoseph Barash GB, Nir Friedman. Algorithms in Bioinformatics, First International Workshop, WABI 2001, Aarhus, Denmark, August 28-31, 2001, Proceedings. In: Moret OGaBME, editor. Lecture Notes in Computer Science; 2001 2001; Aarhus, Denmark. Springer. pp. 278-293.

13. Wang W, Grimmer JF, Van De Water TR, Lufkin T (2004) Hmx2 and Hmx3 homeobox genes direct development of the murine inner ear and hypothalamus and can be functionally replaced by Drosophila Hmx. Dev Cell 7: 439-453.

14. Wang W, Lufkin T (2005) Hmx homeobox gene function in inner ear and nervous system cell-type specification and development. Exp Cell Res 306: 373-379.
