# Supplementary material for: A cis-Regulatory Signature for Chordate Anterior Neuroectodermal Genes
Source: PLoS Genet. 2010 Apr 15;6(4):e1000912. doi: 10.1371/journal.pgen.1000912 (PMC2855326; doi:10.1371/journal.pgen.1000912)
Supplement: Table S2 — Annotation of expression patterns for 258 genes at early-/mid-tailbud stages. (1.40 MB PDF) [file pgen.1000912.s006.pdf]

Table S2

No symbols could be identified with BLAST for these genes

Gene	stomodeum	tip.of.ant.sens.ves	ant.sens.ves	posterior.sens.ves	palps	visceral.ganglion	neck	nerve.cord	nervous.system
ALX4 (36333)		x							
AHR (42195)								x	
AEBP2 (30396)			x	x					
ADAMTS15 (37065)								x	
54565			x	x		x			
54222			x	x					
53667							x		
53163								x	
53128					x				
52773						x			
51908			x	x		x		x	
51882				x		x		x	
51750						x			
51460									
50166			x	x	x			x	
49555			x	x		x			
48961									x
48006								x	
46821									x
46740			x	x		x		x	
46016			x	x		x		x	
45411									x
44588			x						
43866			x	x					
43696			x						
42607		x						x	
42575							x		
40773								x	
40454				x		x			
40350									x
40298		x							
39809								x	
39388						x			
39345								x	
39310					x				
39274			x	x					
38919									x
38905			x	x		x		x	
38545			x	x		x			
38488				x					
38433					x				
38327			x						
37237			x					x	
36307				x					
35421			x						
34992			x	x					
32852			x					x	
32801			x	x		x			
32611								x	
31431			x						
30546					x				
30459									

Gene	stomodeum	tip.of.ant.sens.ves	ant.sens.ves	posterior.sens.ves	palps	visceral.ganglion	neck	nerve.cord	nervous.system
FGF9 (51552)				x			x		
FGF3 (51754)				x		x		x	
FGF13 (34844)									
FABP6 (36755)								x	
EYA1 (37801)	x								
EXOSC4 (31447)					x				
EVI1 (30183)			x	x					
ETS1 (40048)			x			x			
EPHA7 (36022)						x		x	
EPHA4 (41382)									
EPHA4 (36974)	x			x				x	
EPB41L5 (34551)	x								
ENPP3 (41161)								x	
ENPP2 (48528)								x	
EN1 (50641)				x		x			
EFNB3 (48665)	x	x			x				
EFNA4 (51960)	x	x				x		x	
EFNA3 (51929)	x					x			
EDEM3 (37372)			x						
EBF1 (33604)						x			
DUSP16 (33347)		x							
DUSP1 (38796)		x							
DMRTA1 (50302)			x	x					
DLX6 (45240)			x		x				
DLX3 (49957)					x				
DLX1 (49931)	x								
DKK2 (31688)			x	x		x		x	
CXXC4 (40019)								x	
CSMD3 (49322)	x								
CREB1 (52330)	x								
CPLX2 (50132)				x		x			
COL2A1 (50759)								x	
CHRD (50422)									x
CDX1 (48516)								x	
CDC6 (36387)								x	
CDA (49658)			x						
CASZ1 (36802)					x				x
CARS (52787)								x	
CARS (46220)								x	
CARS (36081)								x	
CARS2 (38168)								x	
BSX (46138)		x	x	x					
BRAF (30559)				x					
BMPER (41753)	x								
BMP7 (47380)			x	x	x				
BMP3 (30198)			x						
BCMO1 (41268)			x	x					
ATP6V1H (45745)			x	x		x			
ARNT (43114)									
ARNT (41648)									
ARID3A (30816)	x		x						
ARF1 (31662)								x	
ANKMY2 (32761)			x						

Gene	stomodeum	tip.of.ant.sens.ves	ant.sens.ves	posterior.sens.ves	palps	visceral.ganglion	neck	nerve.cord	nervous.system
LPHN3 (50579)	x	x	x	x					
LMX1A (50069)								x	
LHX3 (30659)						x			
LASS6 (33892)						x			
KLF4 (42734)	x		x	x	x	x		x	
KLF12 (49971)									
ISL1 (34923)			x			x			
ISCA2 (42907)			x	x		x			
IRX6 (31105)						x			
IRX6 (31049)			x			x			
IRX2 (32122)			x			x			
INSR (42945)									x
INSM1 (49176)								x	
IHH (52836)								x	
ICA1 (31810)									x
HSP90AA1 (34890)			x	x					
HOXB5 (44131)								x	
HOXA1 (43927)						x	x	x	
HMGB3 (30770)									x
HMGB2 (30355)									x
HMCN1 (37707)	x	x							x
HMCN1 (31575)					x				
HIF1A (44358)			x						
HES1 (43019)				x	x				
HES1 (39157)	x		x	x		x		x	
H3F3A (31229)									x
GUCY1B2 (42223)		x	x						
GUCY1B2 (31817)			x						
GUCY1A2 (54703)			x						
GTDC1 (32134)								x	
GSX2 (51030)			x	x					
GSK3B (50286)			x						
GSC (33258)				x					
GREM1 (38042)			x						
GOLGA5 (50375)		x	x		x				
GIT1 (30539)								x	
GAK (39892)					x				
GABARAP (36260)			x	x	x	x		x	
FZD4 (53324)								x	
FZD4 (52761)								x	
FTH1 (41760)					x				
FOXP1 (31811)			x						
FOXN3 (30540)			x						
FOXJ3 (49624)			x						
FOXJ3 (48077)			x						
FOXJ1 (44854)	x	x							
FOXI2 (36433)									x
FOXH1 (52814)	x	x	x	x	x				
FOXF2 (37186)	x	x	x	x	x				
FOXD3 (46995)								x	
FOXD3 (42807)				x		x			
FOXC1 (31711)			x		x				
FOXB1 (45669)			x	x					

Gene	stomodeum	tip.of.ant.sens.ves	ant.sens.ves	posterior.sens.ves	palps	visceral.ganglion	neck	nerve.cord	nervous.system
SERPINC1 (32818)			x						
SALL1 (47367)								x	
SALL1 (41112)								x	
RORB (35919)	x								
RHOT1 (51356)								x	
RFX3 (30296)	x	x							
RERG (46313)	x								
RAP1B (37334)								x	
PTF1A (44571)				x					
PRICKLE2 (38921)				x				x	
PRDM16 (30182)			x	x					
PPP5C (34412)			x	x		x			
POU4F2 (46655)		x	x		x				
PIWIL1 (37994)				x					
PITX2 (47848)	x	x							
PIAS2 (39519)			x	x					
PHOX2A (33480)							x		
PHKG2 (40723)				x		x			
PELI1 (51400)					x				
PDE6D (41740)		x	x						
PAX7 (37248)				x		x	x	x	
PAX6 (44072)		x	x			x		x	
OTX2 (33709)	x	x	x	x					
OTP (32453)	x								
OPN1MW (43990)			x	x					
ONECUT2 (39609)			x			x			
NTN1 (45554)				x					
NR6A1 (49164)			x	x		x			
NR1D1 (43297)						x			
NPAS3 (47184)						x			
NOTCH1 (34056)									x
NOG (42187)						x		x	
NKX6−1 (36284)						x		x	
NKX2−1 (43977)				x					
NFKBIA (48369)	x		x	x		x		x	
NEUROG1 (36037)						x			
NDRG3 (44775)			x	x					
MYT1L (31573)			x					x	
MYH10 (43436)					x				
MYCN (50934)	x								
MXD4 (37424)			x	x				x	
MTNR1A (36041)			x						
MTHFD1 (36698)								x	
MSX2 (52602)				x	x	x		x	
MSI2 (41381)				x					
MID2 (37572)	x								
MIB1 (44591)	x		x						
MEX3C (39193)					x				
MEIS1 (30253)			x	x		x		x	
MAPK10 (52355)								x	
MAP2K7 (44731)						x			x
LPHN3 (50579)	x	x	x	x					

Gene	stomodeum	tip.of.ant.sens.ves	ant.sens.ves	posterior.sens.ves	palps	visceral.ganglion	neck	nerve.cord	nervous.system
ZSWIM5 (53738)			x						
ZNRF3 (30630)									x
ZNF643 (47795)					x				
ZNF470 (31913)					x				
ZIC2 (50779)			x					x	
ZFPL1 (51956)				x					
ZFPL1 (32677)				x					
ZEB2 (31373)			x	x		x		x	
ZCCHC11 (50200)								x	
WNT7B (54373)								x	
WNT6 (49085)			x					x	
UNCX (30289)			x	x		x		x	
UBE2S (32491)								x	
TUBB2C (35737)					x				x
TUBA3C (37725)									x
TUBA3C (35224)									x
TUB (31237)									x
TRIM9 (32874)				x					
TRIM36 (54785)			x						
TRAF4 (45911)			x	x		x		x	
TOX (30176)					x			x	
TNRC4 (47349)									x
TNNT2 (46672)			x	x		x			
TLL1 (39988)						x		x	
TH (43675)			x	x					
TGFBR1 (44586)								x	
TFEB (45386)			x			x		x	
TFAP2A (31418)					x				
TEGT (46179)								x	
TEAD1 (54686)	x								
TDH (52576)	x		x						
TCF7L2 (31330)				x					
TBX3 (38873)			x					x	
TBK1 (37807)			x						
TARDBP (51292)								x	
SVIL (35522)	x		x						
STAT5A (31956)			x						
SSTR2 (43330)			x						
SPIC (40005)			x						
SOX1 (48766)		x				x		x	
SMG7 (49075)					x				x
SMAD7 (41647)					x				
SLC6A7 (38200)	x								
SLC6A6 (37997)			x	x				x	
SLC4A7 (33161)		x							
SLC4A7 (30036)		x							
SLC39A2 (35938)			x	x					
SIX3 (53410)	x	x							
SIX2 (43295)	x	x							

Red rectangles on this table indicate an expression in the corresponding tissue as annotated in this study.
All annotations are based on in-situ images download ed from the Aniseed Database (http://crfb.univ-mrs.fr/aniseed/).
Gene symbols are based on the best BLAST match in the human proteome
Numbers in brackets refer to JGI1 gene model IDs. For brevity, we have omitted the leading ci 01001xxxxx e.g. the gene ID for FGF9 is ci0100151552
